# Genetic resistance and specificity in sister taxa of *Daphnia*: insights from the range of host susceptibilities

**DOI:** 10.1186/s13071-019-3795-y

**Published:** 2019-11-20

**Authors:** Sigal Orlansky, Frida Ben-Ami

**Affiliations:** School of Zoology, George S. Wise Faculty of Life Sciences, Tel Aviv University, 6997801 Tel Aviv, Israel

**Keywords:** *Daphnia magna*, *Daphnia similis*, Disease trait expression, Genotype-by-genotype (G×G) interactions, *Hamiltosporidium*, Parasite transmission, Virulence

## Abstract

**Background:**

Host genetic diversity can affect various aspects of host-parasite interactions, including individual-level effects on parasite infectivity, production of transmission stages and virulence, as well as population-level effects that reduce disease spread and prevalence, and buffer against widespread epidemics. However, a key aspect of this diversity, the genetic variation in host susceptibility, has often been neglected in interpreting empirical data and in theoretical studies. *Daphnia similis* naturally coexists with its competitor *Daphnia magna* and is more resistant to the endoparasitic microsporidium *Hamiltosporidium tvaerminnensis*, as suggested by a previous survey of waterbodies, which detected this parasite in *D. magna*, but not in *D. similis*. However, under laboratory conditions *D. similis* was sometimes found to be susceptible. We therefore asked if there is genetic variation for disease trait expression, and if the genetic variation in disease traits in *D. similis* is different from that of *D. magna*.

**Methods:**

We exposed ten clones of *D. similis* and ten clones of *D. magna* to three isolates of *H. tvaerminnensis*, and measured infection rates, parasite-induced host mortality and parasite spore production.

**Results:**

The two *Daphnia* species differ in the range and variation of their susceptibilities. The parasite produced on average two-fold more spores when growing in *D. magna* clones than in *D. similis* clones.

**Conclusions:**

We confirm that *D. similis* is indeed much more resistant than *D. magna* and suggest that this could create a dilution effect in habitats where both species coexist.

## Background

Parasites are an integral component of ecological communities [1]. Host-parasite interactions influence a variety of ecological and evolutionary processes [2, 3] and in return these interactions are influenced by other organisms in the habitat such as other parasites, other hosts and predators [4, 5]. The resulting disease dynamics are also affected by host genetic variation in disease traits, such as host susceptibility, virulence and parasite fitness [6, 7]. For example, genotype-by-genotype (G×G) interactions between hosts and parasites can have individual-level effects on parasite infectivity [8], production of parasite transmission stages and virulence [9], as well as population-level effects that reduce disease spread [10, 11] and prevalence [12], and buffer against widespread epidemics [13, 14].

One of the key traits by which hosts vary genetically is host susceptibility. If hosts are more susceptible, disease will spread in a population faster and be more widespread, albeit in case of very high virulence, infected hosts may die before they are able to infect other hosts. Notwithstanding, epidemiologists and theoretical ecologists have often neglected variation in host susceptibility when modeling disease spread [15]. For example, regardless of whether transmission is density- or frequency-dependent, in many epidemiological models the susceptibility component of the transmission coefficient is assumed to be invariable within the population [15]. Furthermore, depending on the infection model (gene-for-gene *vs* matching alleles), some studies suggested that genetic variation in host susceptibility would not affect disease spread [14, 16], while others found that it would reduce the risk of disease spread [17, 18]. Even in models that include variable susceptibility, both average susceptibility and variation in susceptibility are themselves likely to vary with host density and the availability of host resources [15], e.g. density-dependent prophylaxis [19]. Most of our knowledge about variation in host susceptibility comes from studies of host species in which there are both susceptible and resistant clones/genotypes within the population. Little is known about the variation in host susceptibility (or lack of it) in relatively resistant host species, i.e. species that are rarely or even never found to be infected by parasites (endo- or ectoparasites).

*Daphnia magna* Straus and *Daphnia similis* Claus are closely related (sister taxa) freshwater planktonic crustaceans that reproduce *via* cyclical parthenogenesis. They are often found in sympatry in pools around the Mediterranean Sea, and have a largely overlapping geographical distribution in Eurasia [20, 21], including Israel [22]. However, while *D. magna* is host to a variety of parasites [23, 24], a survey of 22 waterbodies in Israel did not detect any endo- or ectoparasites in *D. similis*, even though other sympatric crustaceans were found to be infected in those habitats [22]. *Daphnia similis* and *D. magna* coexist in about a quarter of these 22 waterbodies [22]. Although we never found infected *D. similis* in the field, under laboratory conditions *D. similis* was sometimes found to be susceptible to *Hamiltosporidium tvaerminnensis*, the parasite used in the present study (F. Ben-Ami and S. Orlansky, unpublished data). We therefore asked if genetic variation exists in disease traits (i.e. host susceptibility, parasite-induced host mortality, parasite fitness) among *D. similis* clones, similar to the variation observed in *D. magna* [25, 26]. Our results indicate that the two *Daphnia* species differ in the range and variation of their susceptibilities. However, there is no evidence of genetic variation in parasite-induced host mortality and parasite spore production among *D. similis* clones.

## Methods

We used six *D. magna* clonal lines (genotypes) from Israel, two *D. magna* clones from central Europe and two *D. magna* clones from northern Europe. Ten *D. similis* clones were sampled in Israel. All Israeli *D. magna* and *D. similis* clones originated from separate waterbodies in geographically diverse locations up to 140 km apart. The 20 clones are listed in Table 1. Due to the ecological and biogeographic similarities between the two *Daphnia* species, in this study we used three *Hamiltosporidium tvaerminnensis* isolates, two from Israel and one from northern Europe. *Hamiltosporidium tvaerminnensis* (formerly *Octosporea bayeri*) is an obligate intracellular microsporidium [27, 28] that is known to infect *D. magna* in various locations across Europe and Israel [22, 29].

**Table 1:** List of clones of *Daphnia* species used in this study

Species	Clone	Origin	Location (Region) in Israel
*D. magna*	FI-N-47-6	Finland	
*D. magna*	SE-G2-8	Sweden	
*D. magna*	HU-HO2	Hungary	
*D. magna*	BE-M10	Belgium	
*D. magna*	IL-SK-2	Israel	Hula Valley
*D. magna*	IL-HSN-2	Israel	Haspin North (Golan Heights)
*D. magna*	IL-HSS-1	Israel	Haspin South (Golan Heights)
*D. magna*	IL-BS-1	Israel	Bar-On (Golan Heights)
*D. magna*	IL-NA-1	Israel	Naaman (Northern Coastal Plain)
*D. magna*	IL-PS-2	Israel	Poleg (Central Coastal Plain)
*D. similis*	IL-Sim-A20	Israel	Maskana (Galilee)
*D. similis*	IL-DSKYN-2	Israel	HaKfar HaYarok (Central Coastal Plain)
*D. similis*	IL-DSKYN-3	Israel	HaKfar HaYarok (Central Coastal Plain)
*D. similis*	IL-DSKYN-4	Israel	HaKfar HaYarok (Central Coastal Plain)
*D. similis*	IL-DSZ-2	Israel	Zarta (Samaria)
*D. similis*	IL-DSB-3	Israel	Bareket (Samaria)
*D. similis*	IL-DSB-6	Israel	Bareket (Samaria)
*D. similis*	IL-DSN-2	Israel	Nizanim (Southern Coastal Plain)
*D. similis*	IL-DSN-3	Israel	Nizanim (Southern Coastal Plain)
*D. similis*	IL-DSNS-1	Israel	Nizanim (Southern Coastal Plain)

We conducted an infection experiment with 20 host clones and three parasite isolates (plus controls) to test for resistance against *H. tvaerminnensis*. Prior to the experiment and to minimize maternal effects, third-generation mothers from each *Daphnia* species and clone (separate maternal lines) were kept in 400-ml jars with 10–12 individuals in each jar. We then followed a cohort of 440 *D. magna* individuals (10 clones × 3 parasite isolates × 12 replicates = 360, plus 10 clones × 8 replicates for the controls = 80) and 440 *D. similis* individuals. The cohort consisted of newborns (0–48 hours-old) that were separated from the mother generation and fed with 1 × 10^6^ *Scenedesmus* sp. algae cells per day per *Daphnia*. To accommodate the growing food demands, on days 9, 15, 18, 22 and 27, we increased the daily food level for all individuals to 3 × 10^6^, 5 × 10^6^, 6 × 10^6^, 7 × 10^6^, 8 × 10^6^ algae cells per day, respectively. On day 6, individuals were exposed (controls were sham exposed) to approximately 300,000 spores of the respective parasite isolate, and individually placed in jars filled with 20 ml of artificial medium [30, 31]. After a week, *Daphnia* were transferred to 100-ml jars filled with fresh artificial medium and thereafter artificial medium was replaced whenever the animals reproduced. The temperature was kept at 21 ± 0.5 °C and a light: dark cycle of 16 h: 8 h. All treatments were randomly distributed on the shelves and rearranged often to prevent position effects. Dead animals were recorded daily, but only animals that had died after day 14 were scored for infection under a phase contrast microscope (200–400×), because animals that had died earlier could not be reliably scored for infection [32, 33]. Thereafter dead animals were frozen in 1 ml of artificial medium at − 20 °C for subsequent parasite spore counting using a haemocytometer (Thoma ruling).

### Statistical analysis

All statistical tests were carried out using R, version 3.5.1 (R Core Team, www.R-project.org). Infectivity was analyzed using binary logistic regression (proc glm, family = binomial), with host species, host clone and parasite isolate coded as indicator variables. Cox regression (proc coxph) was used in a similar way to compare parasite-induced host mortality (virulence) among treatments, with time-to-host-death-since-exposure as the dependent variable. The effects of host species, host clone, parasite isolate and their interactions on parasite spore production were examined using a general linear model (proc glm, family = quasi). Tukey contrasts with Bonferroni-adjusted *P*-values were used in multiple comparisons of parasite-induced host mortality (proc glht).

## Results

### Host susceptibility and parasite infectivity

**Fig. 1:**
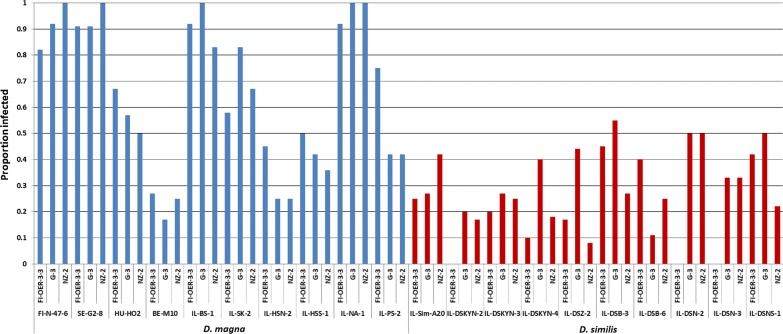
Proportion infected in each host clone-parasite isolate combination for *D. magna* and *D. similis*

**Fig. 2:**
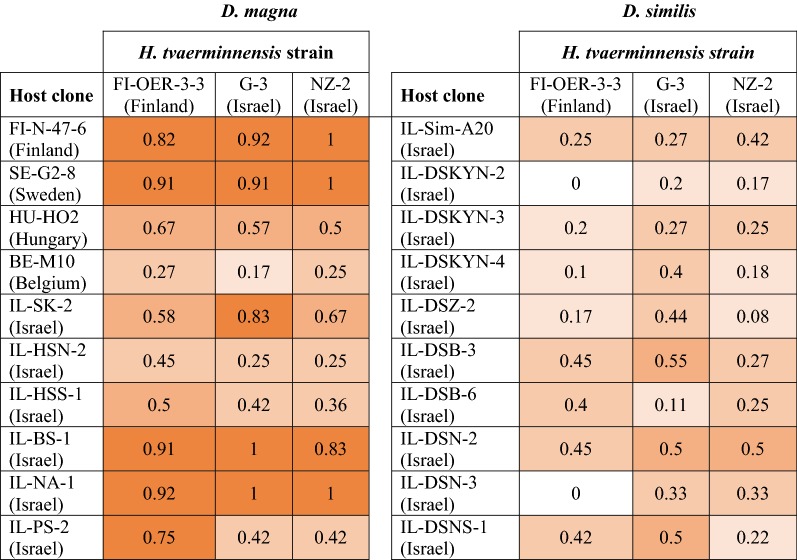
Infection heat map for *D. magna* and *D. similis*

Overall, *D. magna* clones were more susceptible to infection than *D. similis* (binary logistic regression, *z* = − 8.96, *P* < 0.0001; Table 2), regardless of parasite isolate (*P* > 0.32) and host species by parasite isolate interactions (*P* > 0.18). The proportion of infected *D. magna* clones ranged from 17 to 100%, while it ranged from 0 to 55% in *D. similis* (Figs. 1 and 2), with host clone, but not parasite isolate, significantly affecting infection rates (Table 3). The wider range of parasite infectivity in *D. magna* was not due to the inclusion of the central and northern European clones (host clones FI-N-47-6, SE-G2-8, HU-HO2 and BE-M10 in Figs. 1 and 2), i.e. excluding the European clones did not alter the range of parasite infectivity. Furthermore, infection rates of all *Daphnia* clones as well as only Israeli clones differed between species (all clones: *F*_(1, 58)_ = 38.8, *P* < 0.0001; Israeli clones: *F*_(1, 46)_ = 34.0, *P* < 0.0001).

**Table 2:** Mean ± SE of various disease traits by parasite isolate

Disease trait	*D. magna*			*D. similis*		
	G-3	NZ-2	FI-OER-3-3	G-3	NZ-2	FI-OER-3-3
Host susceptibility (proportion)	0.65 ± 0.10	0.63 ± 0.10	0.68 ± 0.07	0.36 ± 0.05	0.27 ± 0.04	0.25 ± 0.05
Virulence (days)	65.2 ± 3.6	57.7 ± 3.3	59.7 ± 3.3	52.9 ± 4.4	66.8 ± 4.6	68.2 ± 4.4
Parasite fitness (spores, log-transformed)	4.95 ± 0.45	5.01 ± 0.30	4.86 ± 0.06	3.16 ± 0.01	3.14 ± 0.01	2.52 ± 0.01

*Note*: Host longevity of control *D. magna* and control *D. similis* was 86.1 ± 3.6 days and 73.7 ± 2.9 days, respectively

**Table 3:** Binary logistic regression analysis of the effects of host clone and parasite isolate on the infection status of *D. magna* and *D. similis*

Independent variable	*D. magna*			*D. similis*		
	LR	*df*	*P*	LR	*df*	*P*
Host clone	119.83	9	**< 0.0001**	16.98	9	**0.049**
Parasite isolate	0.67	2	0.71	1.81	2	0.40
Host clone * Parasite isolate	13.85	18	0.74	19.70	18	0.35

*Abbreviations*: LR, likelihood ratio; df, degrees of freedom

*Note*: Bold typeface indicates significant effect

### Parasite-induced host mortality (virulence)

**Fig. 3:**
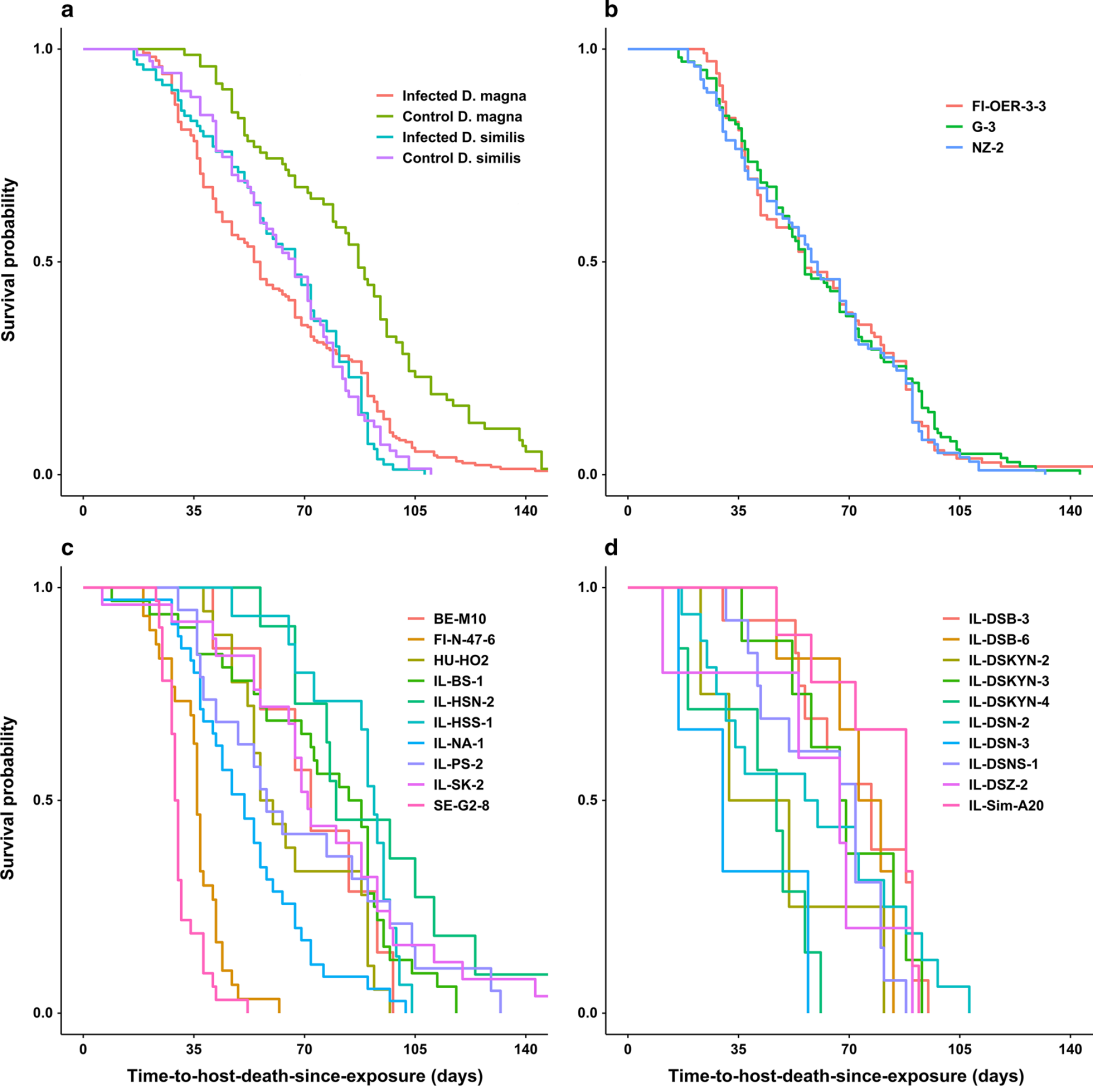
Time-to-host-death-since-exposure by host species (infected and control groups) (**a**), parasite isolate (**b**), *D. magna* clones (**c**), *D. similis* clones (**d**)

Host mortality in control *D. magna* was lower than in control *D. similis* (Cox regression hazard ratio = 2.69, *z* = 5.36, *P* < 0.0001; Table 2). However, there was no difference in the overall mortality of infected *D. magna vs* infected *D. similis* for all parasite isolates (Table 4, Fig. 3a, b). Infected *D. magna* clones differed from each other in their mortality (Tukey contrasts with Bonferroni-adjusted *P*-values: *z* = − 9.81–9.84, *P* = 2e−16–0.026; Fig. 3c) and from the control group (*z* = − 4.96, *P* < 0.0001). For *D. similis* there were no differences in mortality among clones (Tukey contrasts with Bonferroni-adjusted *P*-values: *z* = − 2.99–2.70, *P* > 0.12; Fig. 3d), and no difference between infected and control animals (*z* = − 0.39, *P* = 0.70).

**Table 4:** Cox regression analysis of the effects of host species and parasite isolate on time-to-host-death-since-exposure (virulence)

Independent variable/contrast	HR	z	*P*
Host species	1.13	0.91	0.36
Parasite isolate G-3 *vs* FI-OER-3-3	0.95	− 0.34	0.73
Parasite isolate NZ-2 *vs* FI-OER-3-3	1.06	0.38	0.71

*Note*: Host species by parasite isolate interactions were not significant

*Abbreviation*: HR, hazard ratio

### Parasite spore production (parasite fitness)

**Fig. 4:**
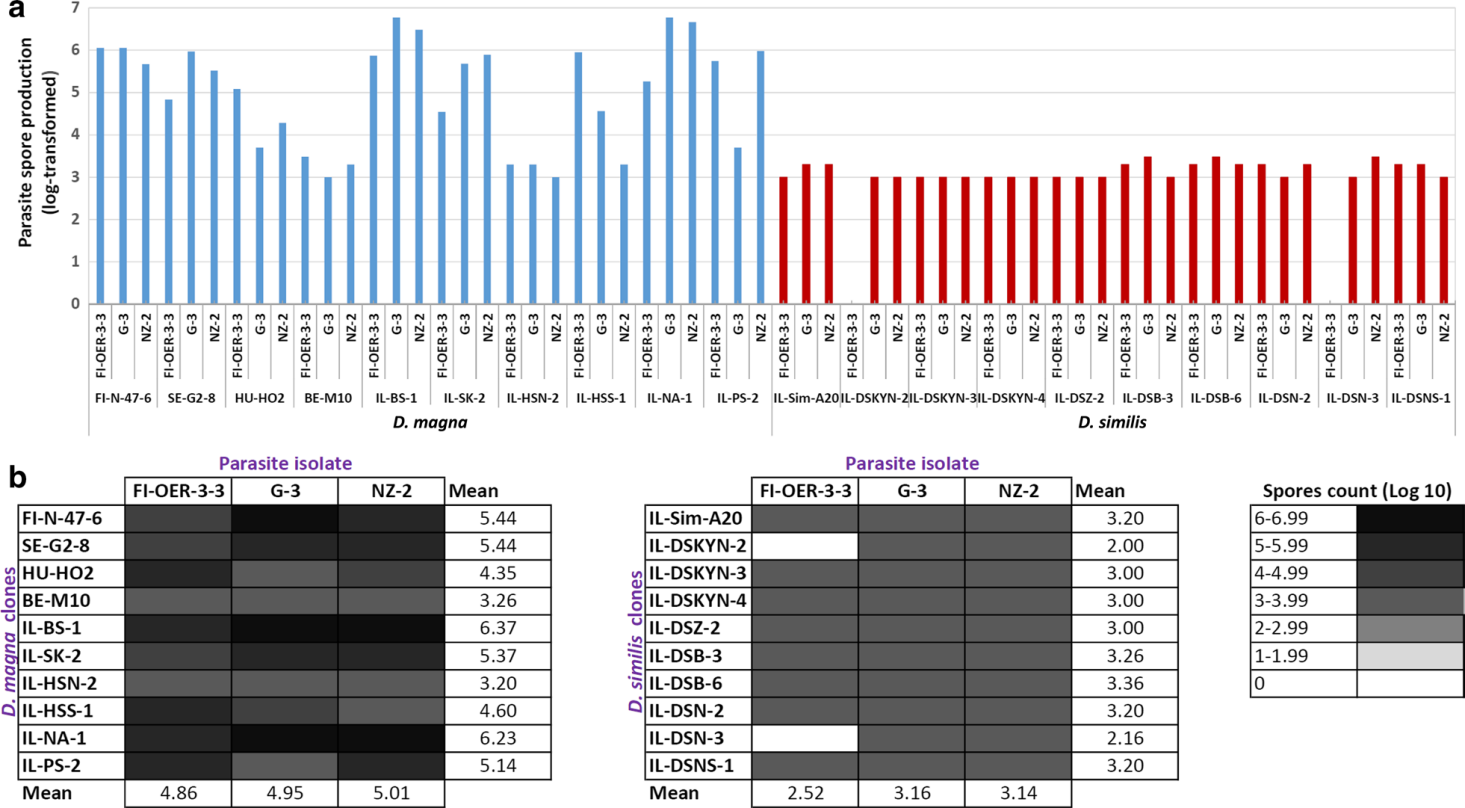
Parasite spore production (log-transformed) in each host clone-parasite isolate combination for *D. magna* and *D. similis*: **a** bar graph **b** matrix

The parasite produced on average two-fold more spores when growing in *D. magna* clones than in *D. similis* clones (*z* = − 9.49, *P* < 0.0001; Table 2, Fig. 4a, b). Parasite spore production differed among *D. magna* clones, but not among *D. similis* clones (Table 5). Furthermore, when infecting *D. magna*, no differences in spore production were found between the European isolate and the Israeli isolates (FI-OER-3-3 *vs* G-3: *z* = 0.15, *P* = 0.23; FI-OER-3-3 *vs* NZ-2: *z* = 0.16, *P* = 0.94; G-3 *vs* NZ-2: *z* = 0.15, *P* = 0.78). However, when infecting *D. similis*, the European isolate produced fewer spores than both Israeli isolates did, while no difference in spore production was found between the two Israeli isolates (FI-OER-3-3 *vs* G-3: *z* = 6.23, *P* < 0.0001; FI-OER-3-3 *vs* NZ-2: *z* = 5.09, *P* < 0.0001; G-3 *vs* NZ-2: *z* = − 1.04, *P* = 0.90).

**Table 5:** Quasi-Poisson regression analysis of the effects of host clone and parasite isolate on parasite spore production of *D. magna* and *D. similis*

Independent variable	*D. magna*			*D. similis*		
	LR	*df*	*P*	LR	*df*	*P*
Host clone	93.07	9	**<** **0.0001**	6.60	9	0.68
Parasite isolate	27.16	2	**<** **0.0001**	0.90	2	0.64
Host clone * Parasite isolate	51.05	18	**<** **0.0001**	6.32	15	0.97

*Abbreviations*: LR, likelihood ratio; df, degrees of freedom

*Note*: Bold typeface indicates significant effect

## Discussion

Consistent with field data that suggested that *D. similis* has a high level of parasite resistance [22], our experiment in the laboratory revealed high levels of resistance, as compared to the more susceptible host *D. magna*. Although host mortality of control *D. magna* was lower than that of control *D. similis*, there was no difference in parasite-induced host mortality between infected *D. magna* and infected *D. similis*. In comparison with *D. magna*, infected *D. similis* produced fewer parasite transmission stages and there was no evidence of genetic variation in parasite-induced host mortality and parasite spore production among *D. similis* clones.

Our finding that the range of host susceptibilities of the resistant host *D. similis* was lower than that of the susceptible host *D. magna* might be related to the origin of the *Daphnia* clones. While all ten *D. similis* clones originated from Israel, four of the ten *D. magna* clones originated from central or northern Europe and the other six from Israel. Cladoceran habitats in the Levant (a stretch of land adjacent to the eastern shore of the Mediterranean Sea, about 800 km long and approximately 150 km wide [20, 22, 34]) differ from central and northern European habitats, because they are summery-dry, undergo a planktonic phase in winter, do not freeze and have no fish predation (due to being summery-dry). Nevertheless, infections with the European *D. magna* clones included both highly resistant and highly susceptible host clone-parasite isolate combinations, very much like the six Israeli *D. magna* clones (Figs. 1 and 2). Lange et al. [35] found that multi-generation, long-term persistence of *H. tvaerminnensis* in monoclonal populations of *D. magna* was only possible in hosts collected from their natural geographical range. They further showed that the genetic distance between hosts from the parasite’s origin site and naïve host populations correlated negatively with parasite persistence [35]. Although Lange et al. [35] excluded environmental variation in their experiments, they suggested that the parasite persisted only in host populations from summery-dry habitats, which are also widespread in Israel. Given that six out of ten *D. magna* clones and all ten *D. similis* clones originated from geographically diverse locations across Israel, it is likely that the variation in susceptibility of both host species had a genetic rather than a geographical basis. However, further studies are needed to disentangle among genetic, ecological and geographical covariables, in order to explain the range and variation of host susceptibilities in these sister taxa of *Daphnia*.

Our finding that *D. similis* has a high level of parasite resistance in comparison to *D. magna*, despite the widespread abundance of the latter species throughout Eurasia, may be suggestive of parasite-mediated interspecific competition, especially since coexistence of both *Daphnia* species was found by Goren & Ben-Ami [22]. Parasites can be instrumental in mediating interspecific competition between host species [36–38]. Their influence may be direct, e.g. by reducing the density or competitive strength of an otherwise competitively superior host in interactions between two host species or between host and non-host species [39–41]. Their influence may also be indirect [42], e.g. infections of the dominant herbivorous snail *Littorina littorea* by the digenean trematode *Cryptocotyle lingua* along the northern Atlantic coast of North America reduced its grazing rate and thus indirectly affected the composition of the macroalgal community [43]. Population-level experiments are needed to assess the role of parasites in mediating interspecific competition between *D. similis* and *D. magna*.

Successful infection requires some degree of genetic compatibility between host and parasite genotypes. The matching-alleles (MA) model, mainly championed by invertebrate zoologists [44, 45], assumes a symmetric match between host and parasite alleles, similar to self-nonself recognition systems found in animal immune systems [46]. It has been shown that the resistance of *D. magna* against the bacterium *Pasteuria ramosa* follows the MA model [47]. Our findings are consistent with the MA model, as some *D. magna*-*H. tvaerminnensis* combinations (see also [26]) and some *D. similis*-*H. tvaerminnensis* combinations were more compatible than others were (Figs. 1 and 2). Although host clone by parasite isolate interactions in infectivity were not statistically significant in both host species (Table 3), there was no single host clone that was superior to all other clones in the resistance to every parasite isolate (Figs. 1 and 2). Likewise, there was no parasite isolate that was superior to all other isolates in infectivity to every host clone (Figs. 1 and 2). Moreover, infections of *D. similis* by the European *H. tvaerminnensis* isolate resulted in the production of fewer parasite transmission stages compared with infections by Israeli parasite isolates (Fig. 4b), which is suggestive of parasite local adaptation, albeit no such differences between the European and Israeli isolates were found in *D. magna* infections (Fig. 4a).

Host genetic diversity has been suggested as a defense mechanism against the spread of infectious diseases [48, 49]. Experimental studies that quantified the effects of genetic variation on resistance against parasites in relatively susceptible hosts, found that parasites spread significantly faster in host populations of low diversity compared to host populations of high diversity [50–52], regardless of parasite diversity [53]. Furthermore, parasite prevalence was lower in genetically variable host populations [50–52]. Van Baalen & Beekman [54] argued for an additional precondition that genetically diverse host populations are susceptible to a larger suite of parasites. They further argued that although population variability reduces the expected costs of infection, this might not be sufficient for a genetically heterogeneous group to offset the increased rate of acquiring infection, which leads to a subtle balance of costs and benefits associated with host heterogeneity. *Daphnia similis* has never been reported to be infected by any microparasites [22] and laboratory attempts to infect *D. similis* with another parasite species have not been successful [55]. This might suggest that genetic diversity is less advantageous for relatively resistant host populations. However, to ascertain the role of genetic diversity in the resistance of *D. similis*, it would be necessary to determine how diverse are *D. similis* populations in comparison with *D. magna* populations, especially in waterbodies where both species coexist.

Parasite spore load in *D. similis* individuals was on average more than two-fold lower than in *D. magna* individuals, regardless of the parasite isolate’s origin. Although *H. tvaerminnensis* can infect its host both horizontally and vertically (mixed-mode transmission; [56]), only horizontal transmission can infect other host species. Parasite spore load in horizontal transmission is used as an estimate of transmission potential, as it often correlates with parasite transmission rate [57–59]. Additionally, the duration of infection was similar, as we found no difference in parasite-induced host mortality between infected *D. magna* and infected *D. similis*. Taken together, infections by *D. similis* could cause a dilution effect in terms of the number of parasite spores released into the environment in a given period. This dilution effect is in addition to dilution *via* removal of parasite spores without becoming infected [60]. Additionally, the observed patterns of differential susceptibility of *D. similis vs D. magna* could feedback to affect parasite transmission [61]. Therefore, *D. similis* may benefit *D. magna* and contribute to epidemic fadeout when they coexist in the same pond or rain pool.

The dilution effect has attracted considerable attention among evolutionary ecologists, as it links between host communities and disease transmission [62]. The successful outcome of dilution among competitors depends on three prerequisites: encounter reduction (i.e. removal of parasite spores without becoming infected), the magnitude of disease spread and the strength of competition [63]. Since *Daphnia* species feed on particles in the size range of parasite spores [23, 64], the spores may either cause an infection or be destroyed in the host gut, but see [65] for a case where spores survived gut passage. In our study system, the diluter *D. similis* may remove parasite spores from the environment as well as reduce the number of parasite spores released into the environment in a given period. Thus, the first prerequisite for successful dilution is met. The second prerequisite is also plausible, because *D. magna* epidemics are known to be large, with infection prevalence in natural populations varying widely and sometimes reaching 100% [66, 67], including for the here-studied parasite *H. tvaerminnensis* [68]. However, *D. magna* is the most abundant cladoceran in pond environments, whereas *D. similis* is less often found, only in 27% of cases together with *D. magna* [22]. Thus, *D. magna* appears to be a stronger competitor, making it unlikely that *D. similis* depresses *D. magna* density by depleting shared resources—the third prerequisite of successful dilution. It remains to be determined how these three prerequisites interact and affect the success of dilution in the *D. magna*-*D. similis* species complex.

The differential parasite spore load as well as the variation in parasite-induced host mortality among *D. magna* clones support the conjecture that increased parasite spore load induces mortality on infected host and increases horizontal transmission, or is indicative of horizontal transmission efficacy [69]. In contrast with *D. magna*, parasite proliferation in *D. similis* was low and seemed not to affect host survival, as infected hosts did not die earlier than the control group.

## Conclusions

Our findings suggest that the two *Daphnia* species differ in the range and variation of their susceptibilities. The parasite produced on average two-fold more spores when growing in *D. magna* clones than in *D. similis* clones. We confirm that *D. similis* is indeed much more resistant than *D. magna* and suggest that this could create a dilution effect in habitats where both species coexist. Our results emphasize that the specificity of *D. similis* resistance has the potential to maintain genetic diversity in both host and parasite populations. Such specificity can shape the ecology and evolution of infectious disease in pond habitats where both *Daphnia* species coexist. Future studies should unravel the mechanism driving exclusion (e.g. interspecies competition, parasitism) and coexistence in the *D. magna*-*D. similis* species complex.

## Data Availability

The datasets used and/or analyzed during the current study are available from the corresponding author upon reasonable request.
